# *Mycobacterium avium-intracellulare *cellulitis occurring with septic arthritis after joint injection: a case report

**DOI:** 10.1186/1471-2334-7-9

**Published:** 2007-02-26

**Authors:** David M Murdoch, Jay R McDonald

**Affiliations:** 1 School of Public Health, CB#7435, McGravran – Greenberg Hall – 2104H, University of North Carolina at Chapel Hill, Chapel Hill, NC 27599 USA; 2 Division of Pulmonary & Critical Care Medicine, Box 3221, Duke University Medical Center, Durham, NC 27710 USA; 3 Division of Infectious Diseases, Box 3824, Duke University Medical Center, Durham, NC 27710 USA

## Abstract

**Background:**

Cellulitis caused by *Mycobacterium avium-intracellulare *has rarely been described. *Mycobacterium avium-intracellulare *is a rare cause of septic arthritis after intra-articular injection, though the causative role of injection is difficult to ascertain in such cases.

**Case presentation:**

A 57-year-old with rheumatoid arthritis treated with prednisone and azathioprine developed bilateral painful degenerative shoulder arthritis. After corticosteroid injections into both acromioclavicular joints, he developed bilateral cellulitis centered over the injection sites. Skin biopsy showed non-caseating granulomas, and culture grew *Mycobacterium avium-intracellulare*. Joint aspiration also revealed *Mycobacterium avium-intracellulare *infection.

**Conclusion:**

Although rare, skin and joint infections caused by *Mycobacterium avium-intracellulare *should be considered in any immunocompromised host, particularly after intra-articular injection. Stains for acid-fast bacilli may be negative in pathologic samples even in the presence of infection; cultures of tissue specimens should always be obtained.

## Background

*Mycobacterium avium-intracellulare *(MAI) is a common cause of disseminated disease among patients with human immunodeficiency virus (HIV) infection [1], and also occasionally causes pulmonary infections, lymphadenitis, and osteomyelitis in other immunocompromised as well as immunocompetent hosts [2].

Cutaneous disease caused by MAI has a wide variety of clinical presentations, including nodules, pustules, ulcers, abscesses, and draining sinuses [2-4]. Cutaneous MAI usually occurs as a manifestation of disseminated disease. Cellulitis as a primary cutaneous manifestation is rare [4]. MAI septic arthritis has been described in immunocompetent patients [5-7], as well as in patients immunosuppressed by cancer, connective tissue disease, kidney transplant, and HIV [7-10]. MAI septic arthritis has been described after joint injection, however the causative role of the injection is difficult to ascertain [5,10].

We describe a patient with rheumatoid arthritis receiving prednisone and azathioprine who, after corticosteroid injections into both acromioclavicular joints, developed bilateral cellulitis centered over the injection sites and left shoulder septic arthritis.

## Case Presentation

### Clinical history

A 57-year-old Caucasian male was first seen by a rheumatologist in 1998 for arthralgias and myalgias of his proximal upper extremities. His medical history included obesity and hypertension. There was no family history of rheumatologic disease. He worked as a glazier, and had no substance abuse history. Physical examination showed synovitis of his metacarpophalangeal joints and wrists. Erythrocyte sedimentation rate was >120 mm/hr, and rheumatoid factor was negative. He was diagnosed with seronegative rheumatoid arthritis, and prednisone was initiated at 10 mg daily in 1999. His arthralgias and myalgias responded clinically to the prednisone.

In late 1999, because of new symptoms of fatigue, weight loss, and fevers, 10 mg of weekly methotrexate was added to his regimen. An infectious disease consultation was obtained to rule out infection as a cause of his symptoms, and an extensive workup was performed. A chest radiograph was normal, and computed tomography (CT) scans of the abdomen and pelvis were unremarkable. A bone marrow biopsy was normal and cultures for bacteria, mycobacteria, and fungi were negative. Multiple blood cultures for bacteria were negative. With the exception of a persistently elevated sedimentation rate of 133 mm/hr, laboratory results were normal, and no infectious etiology for his symptoms could be found. Because of a lack of response, his methotrexate was discontinued after 3 months of therapy. His weight loss and fevers stopped, and only his fatigue continued.

In January 2002, still on oral prednisone, he was diagnosed with degenerative arthritis of both shoulders. His prednisone was increased to 15 mg daily, and azathioprine 100 mg daily was added. In April 2002, his left acromioclavicular joint was injected with a corticosteroid for symptomatic relief. In June 2002, both acromioclavicular joints were injected with corticosteroids. In December 2002, he first noted an erythematous, warm rash that began simultaneously on both shoulders at the sites of his shoulder injections.

**Figure 1:**
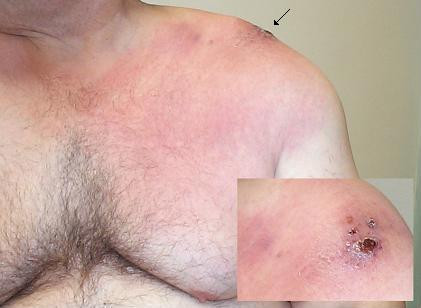
Left chest and shoulder, with arrow indicating site of prior injection into or near the acromioclavicular joint. Insert: Close-up view of area indicated by arrow.

In April 2003, he transferred his rheumatological care to our institution. His immunosuppressant regimen consisted of prednisone 10 mg daily, azathioprine 100 mg daily, and hydroxychloroquine 200 mg twice daily. He complained of fevers, fatigue, worsening bilateral shoulder pain, and progressive bilateral shoulder rash. In addition, he noted new dyspnea without cough. His shoulder pain now prevented him from carrying objects and performing his job. His left shoulder rash, shown in Figure 1, was macular, warm, erythematous, non-tender, and blanched with pressure. At its center was a 5-centimeter crusted ulcer with modest serosanguinous drainage, overlying the acromioclavicular joint. The surrounding erythema had progressed to include his entire left breast to the level of the nipple. It extended slightly onto the left arm and upper back, and did not cross the midline. On the right shoulder, there was a 5-centimeter circular patch of mottled erythema and warmth without ulceration or drainage, overlying the right acromioclavicular joint and symmetric with the central focus of the left-sided rash. There was no appreciable lymphadenopathy. Physical examination of the shoulders was limited by pain with both active and passive range of motion in all directions. There was a palpable effusion of the left shoulder, but not the right. Physical examination of his fingers, wrists, elbows, knees, ankles, and feet was otherwise normal with no evidence of synovitis.

The consulting rheumatologist concurred with the original diagnosis of seronegative rheumatoid arthritis. However, due to the presence of the unexplained rash, alternative and additional diagnoses were considered, including dermatomyositis, sarcoidosis, cutaneous leukemia or lymphoma, and infection. Erythrocyte sedimentation rate was >140 mm/hr, and C-reactive protein titer was 1:16. Anti-nuclear antibodies, uric acid, rheumatoid factor, C3, and C4 were normal. White blood cell count was 13,400 cells/mm^3 ^with a neutrophilic predominance, hemoglobin was 10.6 g/dL and platelet count was 400,000 cells/mm^3^. Serum albumin was 2.8 gm/dL. Serum electrolytes, renal function, liver tests, and creatine kinase were normal. A serum electrophoresis showed polyclonal hypergammaglobulinemia, and ferritin was elevated at 356 ng/ml, suggesting chronic inflammation. A chest radiograph was normal. A skin biopsy of the left shoulder cellulitis in July 2003 revealed non-caseating granulomatous inflammation consistent with sarcoidosis. Stains for fungus and acid-fast bacilli (AFB) were negative, but cultures were not performed.

He was diagnosed with cutaneous sarcoidosis and treated with azathioprine 100 mg daily, prednisone 40 mg daily, and rofecoxib 25 mg daily. Chest CT revealed subtle reticulonodular parenchymal changes with lower lung zone predominance. Though the CT findings were compatible with the diagnosis of sarcoidosis, the lower lobe involvement and absence of lymphadenopathy were atypical.

Because the clinical syndrome was not entirely consistent with sarcoidosis, repeat skin biopsy was performed in September 2003, this time with fungal and AFB cultures in addition to the routine pathology stains. Once again, pathology revealed granulomatous inflammation consistent with sarcoidosis with negative fungal and AFB stains. However, AFB cultures grew mycobacteria after only eight days. The diagnosis of MAI was made by DNA probe, and confirmed by culture. Bacterial and fungal cultures were negative. Serology for HIV was negative.

**Figure 2:**
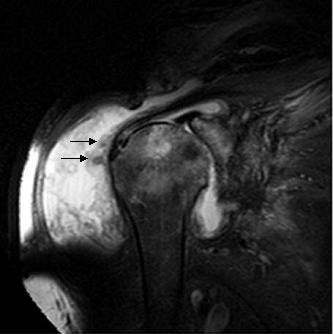
Coronal fat-suppressed T2-weighted magnetic resonance image of the left shoulder revealing diffuse synovitis and bursitis with a large joint effusion. Numerous loose bodies representing rice bodies and degenerative cystic changes within the humeral head were also present (arrow).

Antimicrobial therapy consisting of azithromycin, ciprofloxacin and ethambutol was initiated in December of 2003 by an infectious diseases consultant. Azathioprine was discontinued and corticosteroids were tapered to 7.5 mg po qd, which was the lowest tolerable dose for the patient because of a return of his myalgias and arthralgias. Shoulder plain films and magnetic resonance imaging (MRI) revealed large joint effusions and evidence for avascular necrosis, greater on the left side (Figure 2). Joint aspiration of the left shoulder revealed MAI by culture, and surgical drainage and debridement was performed in January 2004. Intraoperative findings included multiple rice bodies within the joint and a large cloudy effusion. Post-operatively, he was prescribed intravenous amikacin, which was discontinued after 2 weeks due to the development of renal insufficiency. In March 2004, because the cutaneous lesions over his shoulders continued to progress despite initiation of antimicrobial therapy and decrease in immunosuppression, ciprofloxacin was changed to moxifloxacin, and rifabutin was added to his regimen.

The patient completed 21 months of multi-drug therapy with relief but not complete resolution of his rash and shoulder pain. In September 2005, his azithromycin, moxifloxacin, ethambutol, and rifabutin were discontinued on a trial basis because of cumulative drug toxicities. Unfortunately, fevers returned and the rash over the left anterior shoulder worsened. A repeat aspiration of his left shoulder confirmed persistence of MAI septic arthritis with a sensitive isolate. A follow-up CT scan of the chest showed no progression of his previously seen nodules. Antimycobacterial therapy has been re-initiated, and he has undergone a second debridement of his left shoulder. He has since responded to continued antimicrobial therapy documented by a negative joint aspiration, although he continues to have severe arthritic pain from permanent joint damage.

### Mechanisms of introduction

One of two possible mechanisms probably explains the occurrence of MAI in the skin and shoulder. The first possibility is that MAI was introduced directly into the tissue at the time of injection. MAI is ubiquitous in the environment, including water sources, and can contaminate injectable solutions [11]. Because MAI grows slowly, a six-month lag between injection and clinical presentation, as seen in our patient, is biologically plausible. Hoffman et al estimated that 40% of the 19 patients with septic arthritis due to atypical mycobacteria in his series had had previous joint injection; however, only 1 of the injected patients had MAI [12]. It is not reported whether any of the 7 MAI septic arthritis patients in Kozin et al had been previously injected or aspirated [7]. Czachor et al reported a kidney transplant patient who developed MAI septic arthritis months after a diagnostic arthrocentesis for the diagnosis of gout [10]. In our patient, the temporal relationship between the shoulder injections, the subsequent appearance of simultaneous bilateral MAI skin infections at the site of the injections, and the eventual occurrence of left shoulder MAI septic arthritis, provides strong circumstantial evidence for the causative role of joint injection in this patient. Cellulitis of the overlying skin has not been described in previous reports of MAI septic arthritis [6-10,12].

The second possibility is that the patient had undiagnosed disseminated MAI at the time of joint injection, and the trauma of injection caused concentration of the organisms at the injection sites. There is some evidence that this phenomenon occurs in patients with disseminated MAC. Freed et al reported a case of MAI soft tissue infection documented post-mortem at an IV injection site in an HIV- and MAI-infected patient [3]. Meadows et al reported an abscess due to MAI at the site of an intramuscular injection in an HIV-infected patient [13]. Prior to intra-articular injection, our patient had been treated with corticosteroids for approximately 3 years as well as short-term methotrexate and azathioprine. While this degree of immunosuppression makes disseminated MAI possible, there is little evidence to support the theory of pre-existing undiagnosed disseminated MAI infection in our patient, though it remains a possibility. Another possibility may be increased susceptibility to mycobacterial disease due to an underlying genetic defect in the IFN-γ response pathway [14].

## Conclusion

This case illustrates an unusual presentation of MAI: both cellulitis and septic arthritis after bilateral shoulder joint injections. When MAI infects the skin, it typically causes nodules, pustules, and ulcers, but rarely cellulitis [2-4]. It is important to maintain suspicion of atypical mycobacteria in infections of immunocompromised patients, particularly in those undergoing diagnostic and therapeutic procedures. Cultures of biopsied skin should be performed, as special stains for AFB may be negative even in the presence of MAI infection. As with other mycobacteria, the clinical spectrum of MAI is broad, and it should be included in the differential diagnosis of skin and joint infections.

## Competing interests

The author(s) declare that they have no competing interests.

## Authors' contributions

DM conceived the study. Both authors participated in data acquisition and wrote the manuscript. Both authors have seen and approved the final manuscript.

## Pre-publication history

The pre-publication history for this paper can be accessed here:


